# Sharkipedia: a curated open access database of shark and ray life history traits and abundance time-series

**DOI:** 10.1038/s41597-022-01655-1

**Published:** 2022-09-10

**Authors:** Christopher G. Mull, Nathan Pacoureau, Sebastián A. Pardo, Luz Saldaña Ruiz, Emiliano García-Rodríguez, Brittany Finucci, Max Haack, Alastair Harry, Aaron B. Judah, Wade VanderWright, Jamie S. Yin, Holly K. Kindsvater, Nicholas K. Dulvy

**Affiliations:** 1 Integrated Fisheries Laboratory, Department of Biology, Dalhousie University, Halifax, NS B3H 4R2 Canada; 2 Earth to Ocean Research Group, Department of Biological Sciences, Simon Fraser University, Burnaby, BC V5A 1S6 Canada; 3 Department of Fish and Wildlife Conservation, Virginia Polytechnic Institute and State University, Blacksburg, Virginia 24061 USA; 4 Ecology Action Centre, Halifax, NS B3K 4L3 Canada; 5 Departamento de Oceanografía Biológica, Centro de Investigación Científica y de Educación Superior de Ensenada (CICESE), Ensenada, Baja California 22860 Mexico; 6 National Institute of Water and Atmospheric Research (NIWA), Wellington, 6011 New Zealand; 7 14-04 Singapore Business Federation Centre, 068914 Singapore, Singapore; 8 Fisheries & Agriculture Resource Management, Western Australian Government Department of Primary Industries and Regional Development, Hillarys, 6025 Australia; 9 Centre for Sustainable Aquatic Ecosystems, Harry Butler Institute, Murdoch University, Murdoch, 6150 Australia

**Keywords:** Population dynamics, Evolutionary ecology

## Abstract

A curated database of shark and ray biological data is increasingly necessary both to support fisheries management and conservation efforts, and to test the generality of hypotheses of vertebrate macroecology and macroevolution. Sharks and rays are one of the most charismatic, evolutionary distinct, and threatened lineages of vertebrates, comprising around 1,250 species. To accelerate shark and ray conservation and science, we developed Sharkipedia as a curated open-source database and research initiative to make all published biological traits and population trends accessible to everyone. Sharkipedia hosts information on 58 life history traits from 274 sources, for 170 species, from 39 families, and 12 orders related to length (n = 9 traits), age (8), growth (12), reproduction (19), demography (5), and allometric relationships (5), as well as 871 population time-series from 202 species. Sharkipedia relies on the backbone taxonomy of the IUCN Red List and the bibliography of Shark-References. Sharkipedia has profound potential to support the rapidly growing data demands of fisheries management, international trade regulation as well as anchoring vertebrate macroecology and macroevolution.

## Abstract (summary)

**Table:** 

Measurement(s)	Life history traits • Abundance time-series
Technology Type(s)	literature review
Factor Type(s)	Species ID • Location • Year
Sample Characteristic - Organism	Class Chondrichthyes
Sample Characteristic - Environment	All aquatic environments
Sample Characteristic - Location	Global

## Background & Description

“The sea has always challenged [our] minds and imagination and even today it remains the last great frontier of Earth”, a quote from Rachel Carson^1^, speaks to the fascination humans hold for life under the surface, as well as the difficulty in learning its secrets.

Although the sea and its residents are mysterious and diverse, scientific research into the macroecology and macroevolution of fishes has revealed a diversity of life history traits and strategies with deep convergence in their allometric and trait relationships^2,3^. For example, we know that fishes – to a first approximation – are cubes^4^, that there are three main dimensions of fish life histories^5,6^, and that key life history traits such as natural mortality and generation length are shaped by body size and environmental temperature^7–9^. These patterns have been found through comparative analyses of life history trait information (much of which has been compiled in FishBase, one of the first and most extensive bioinformatic databases for fishes^10^). We have learned this wide range of life history traits and strategies underlie a surprisingly narrow range of population dynamics^11–15^. Humans’ long history of exploitation of fishes has yielded numerous time-series of abundance of varying quality that are used, *inter alia* among other things, to develop fisheries stock assessments^16^, track the impacts of climate change^17^, and assess the extinction risk status of species, for example through IUCN Red List of Threatened Species^18^. Time-series of abundance can only be fully understood in the context of life histories. For example, in simple stock assessments the intrinsic population growth rate (*r*) is negatively related to the ecological carrying capacity (*K*), thus requiring an understanding of life histories to refine the set of plausible model scenarios^19,20^. While there are many vertebrate trait databases^21,22^ and some population trend databases^23,24^, we are not aware of any database that offers both traits and trends aligned to the same taxonomy.

For species with high evolutionary diversity and conservation concern, combining information on species traits with information on their population trends through time offer a unique resource for interdisciplinary science^15^. These data are key to prioritizing species for conservation actions through Red List assessments, managing species for sustainable fisheries, developing proposals for listing species on conservation and international trade regulations^18,25,26^. Further, data deficiency is a perennial problem that can be increasingly solved by imputing life histories^27,28^ and ‘borrowing’ information from life histories and time-series of data-rich species to infer the trajectories of data-poor species using ‘Robin Hood’ methods^15,29^. These methods are needed to solve key problems for fish lineages with high risk of extinction that suffer from considerable data paucity, including many shark and ray species^15,30,31^.

Sharks, rays, and chimaeras (class Chondrichthyes, hereafter “sharks and rays”) are one of the three classes of fishes and one of the seven classes of vertebrates^32^. Sharks and rays represent a pivotal point in the evolution and radiation of all vertebrate life for three reasons. First, they are the most evolutionary distinct radiation of vertebrates and represent an important lineage of jawed vertebrates (Gnathostomes) going back ~450 MY^33^. Second, they are the earliest radiation that exhibits the archetype of the vertebrate brain defined by the appearance of the first true cerbellum^34,35^. Third, while sharks and rays do not provide post-partum parental care they exhibit the greatest reproductive diversity among vertebrates; ranging in the degree of maternal investment from egg-laying to live-bearing, including oophagy, intra-uterine cannibalism, and placentotrophy^36,37^. This diversity of morphology, physiology, and life history makes contemporary chondrichthyans an important component of marine ecosystems^38^.

Many unaddressed questions in shark and ray ecology, evolution, and conservation can be best answered with a comparative approach^39^. Motivated by the success of FishBase and the need to continually update the taxonomy of our own personal comparative datasets, we sought a solution to set these data free both to facilitate reproducible science and to make them available for a wider scientific community. Furthermore, more than one third of sharks and rays are threatened with extinction, making them the second most threatened vertebrate lineage after amphibians^33^. Hence, fisheries management and conservation policy processes require access to comparative data to undertake stock assessments, ecological risk assessments, IUCN Red List assessments, and develop advisory materials for conservation policies and agreements^40–42^.

In this Data Descriptor, we introduce Sharkipedia: a curated open-source database designed for the continued updating of life history trait information and population time-series for all sharks and rays across all oceans. Sharkipedia currently hosts information on 58 life history measures related to length (n = 9), age (8), growth (12), reproduction (19), demography (5), and allometric relationships (5) assembled from 264 literature sources. Further, Sharkipedia archives 871 population time-series from 202 species and includes a browser feature to aid the selection of time-series for compilation of biodiversity indicators. This research initiative aims to make all published biological traits and population trends accessible to everyone and accelerate shark and ray research and conservation.

## Methods

**Fig. 1:**
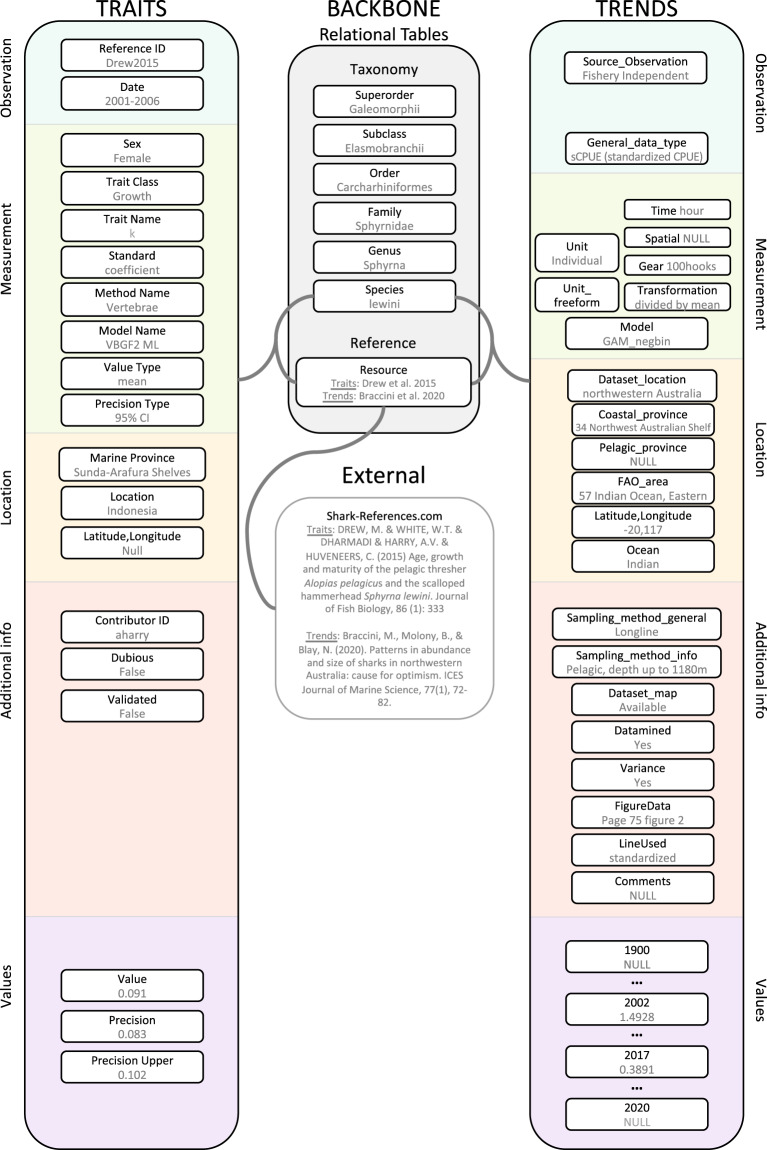
Database Schema. Schema of shared backbone relational tables and Traits and Trends databases. This schema shows a shared example of trait *Measurement* and trend time-series for *Sphyrna lewini* from the Eastern Indian Ocean.

The data are held within the Sharkipedia database (www.sharkipedia.org)^43^, which is designed to hold population/stock level information on the life history traits and population abundance trends for all extant chondrichthyans. The database consists of several associated relational data frames: (1) a structural backbone of chondrichthyan taxonomy and references each uniquely identified by AuthorYear coding and linked to an external valid reference database (the Shark-References database, www.shark-references.com)^44^, (2) life history traits database (i.e. Traits database), and (3) population abundance trends database (i.e. Trends database) (Fig. 1). The most current taxonomic checklist was compiled from a recently published chondrichthyan phylogeny, including recent name changes and species description (www.vertlife.org/sharktree33, global checklists^45^, and the IUCN Red List^40^). The Traits and Trends databases are both related through the taxonomy and reference databases (Fig. 1) so these data streams can easily be brought together for comparative analysis. The specific methods for compiling the traits and trends databases are discussed below.

### Traits

The initial schema for the Traits database (Fig. 1) was developed at a workshop in 2018, bringing together eight experts on shark and ray life history traits. The schema incorporated three requirements for data to be included in the final database: a valid reference ID, valid taxonomic name, and location information. The Traits database contains two core data tables (*Observations* and *Measurements*) used to maintain data traceability, ensure accuracy, avoid duplication, and provide a framework for quality control. *Observations* include metadata associated with a reference–author, year, DOI (Digital Object Identifier)–and are identified with a unique reference code. *Measurements* are tied to the *Observation* and include the information associated with a trait – species, sex, trait class, trait name, value, precision, and method (Fig. 1*Observation* and *Measurement* sections). Location information is coded by Marine Ecoregion^44^ and latitude and longitude when available. Retaining the spatial information for traits will capture interspecific variation in traits with geography. *Measurements* must be coded by ‘trait class’ (e.g., length, age, growth, reproduction, demography, and relationships), which will allow for the selection of appropriate traits (e.g., the “trait class” of growth can be populated by growth model parameters [*k*, L_∞_, L_0_] but not uterine fecundity). Methodological information is retained, for example, for growth trait *Measurements* the model used to estimate parameters is recorded to allow for filtering, selection, or as a covariate in comparative analyses (e.g., two- or three-parameter von Bertalanffy or Gompertz). Estimates of precision (range, standard error, standard deviation) can be recorded when available. Confidence in *Measurements* can be noted when there are known biases, for example incomplete size ranges for age and growth parameter estimation. Finally, new data can be embargoed by researchers prior to publication, to be released by editors upon researcher approval.

### Trends

Time-series data on abundance were gathered from both peer-reviewed publications and grey literature, such as government reports. Further detail on the selection and use of time-series can be found here^18,46^. An *Observation* represents the source of the time-series (fishery-dependent [scientific survey, observer], fisheries, stock-assessment, other) and the trends type (Nominal or Standardized, Catch or Sightings per Unit Effort (CPUE), or abundance, or biomass), where CPUE is ‘Standardized’ by modeling Nominal data with covariates. *Measurement* represents the information associated with trend values – units, transformation, model used – and are tied to the *Observation* (Fig. 1). Location information is coded by Marine Ecoregion^47,48^, FAO Major Fishing Area, Ocean basin, and latitude and longitude (Fig. 3). Location information was entered using the map of the sampled area when available, and indicated in the metadata of the *Observation*. When a map was missing, location was determined by any information found in the text (e.g., latitude/longitude, name of locations, etc.) of the document presenting the data or searched online in the case of scientific survey. Numerical data on abundance are not always reported in documents and are often displayed graphically. We extracted the data from document figures with a digitization software commonly used by scientists (Webplotdigitizer)^49^. The software allows the import of a plot, asks for calibration of axes by clicking known values and then interpolates a coordinate system. Several automatic data extraction functions can be used, and each data point can manually be adjusted (using a magnification between 5 and 10 depending on the plot quality). In addition to trend values, the presence of associated estimates of precision (range, standard error, standard deviation) in the original document were noted.

## Data Records

All datasets are available for viewing, downloading, and contributions through the Sharkipedia database (www.sharkipedia.org) and via Zenodo^50^.

### Traits

**Fig. 2:**
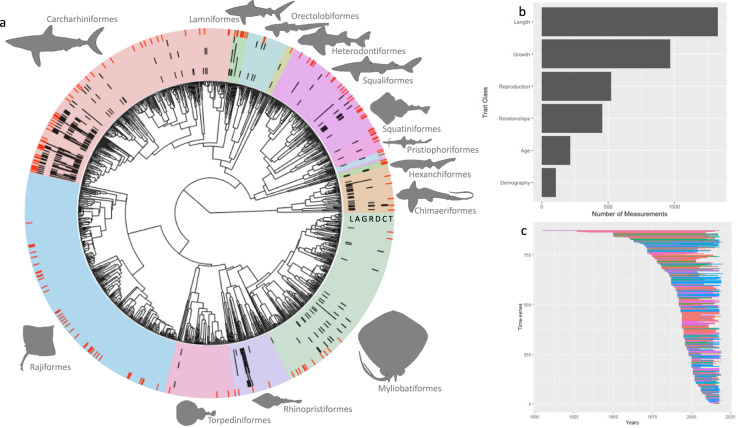
Data Summary. Summary of traits and trends data available in the databases. (**a**) The taxonomic distribution of data on life history traits: (L) Length, (A) Age, (G) Growth, (R) Reproduction, (D) Demography, (C) Relationships, and (T) Trends. (**b**) The number of measurements within each trait class. (**c**) The summary of taxonomy and length of available time-series. Color of time-series indicates taxonomic order corresponding to panel a.

We have compiled data from 264 sources, for 170 species (14% of chondrichthyan diversity) from 39 families (65%) and 12 orders (85%) (Fig. 2a). 155 species contain multiple *Measurements* from distinct populations and/or trait classes. Currently available data is unevenly distributed with more data available for the family of Requiem Sharks (Carcharhinidae) and orders Mackerel Sharks (Lamniformes), and Rhinorays (Rhinopristiformes). Length *Measurements* are the most common type with >1700 currently available, a large proportion of which are the observed maximum length (>700 *Measurements*; Fig. 2b).

### Trends

**Fig. 3:**
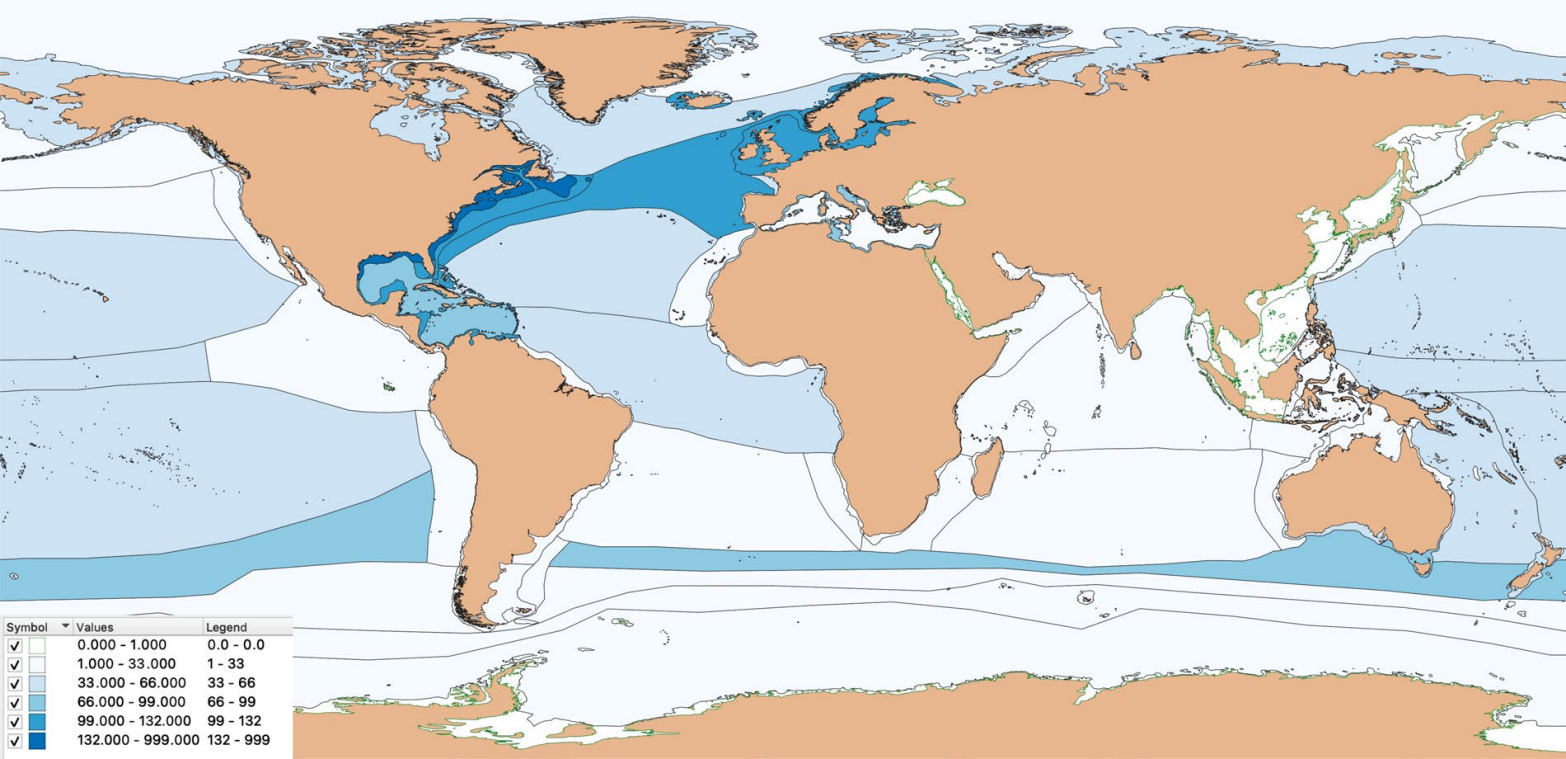
Marine Ecoregions. Abundance time-series available by Marine Ecoregions Of the World.

We have compiled 871 population time-series data from 337 sources (165 published and 172 unpublished), for 202 species (17% of chondrichthyan diversity), from 42 families (70%), and 14 orders (100%) (Fig. 2c). Currently available data is not evenly distributed spatially or taxonomically with more data available in the North Atlantic (e.g., Gulf Stream and North Atlantic Current) and the Southern Subtropical Front, due to the number of time-series from southern Australia (Fig. 3), and more data available for the Carcharhinidae and Rajidae families. Fishery independent data are the most common with 596 time-series currently available, and 64 time-series from stock-assessment (the highest quality of data as it integrates the catch history, abundance trends and life-history information to infer population dynamics).

## Technical Validation

The database is curated on a volunteer basis, including database administrators and editors (https://www.sharkipedia.org/procedure). Volunteer curators are approved based on taxonomic or subject area expertise. Quality control of data includes:

### Contributor approval

To contribute data, users must create a login ID to track data entry. Following sign-up users can contribute data in bulk (e.g., multiple *Observations*) using templates provided to ensure correct data formatting (https://www.sharkipedia.org/imports/new). Single *Observation* entries can be uploaded using the web entry GUI (Traits https://www.sharkipedia.org/observations/new; Trends https://www.sharkipedia.org/trends/new). When data is uploaded, an automated check is conducted to ensure basic requirements are met (valid reference, valid taxon, no required values missing). If basic requirements are not met, the user will be notified immediately of the specific locations (row and cell) of any issues that must be addressed. If basic requirements are met the initial upload is approved.

### Editorial approval

Editors are notified when new *Observations* are uploaded. Editors conduct QA/QC on uploaded data to ensure all data requirements are met, and will check the accuracy of data including location, species, sex, value, and precision. An Editor can reject the upload if there are major issues (e.g., incomplete data entry, missing *Measurements*) with uploaded data. If revisions are required, the Editor can send data back to the contributor with the necessary changes required. Once QA/QC has been completed and all requirements are met the Editor can approve and import the data into the master database. These series of checks ensure that data is thoroughly vetted prior to final import. If issues are noted by users after the final import, core Administrators can manually correct *Measurements*.

### User feedback

Continued population of the database will be Editor and user driven. Users can report any data issues directly to administrators (https://www.sharkipedia.org/contact).

## Usage Notes

The data are available in csv-formatted files and can be accessed via bulk downloads or through species- or trait-specific queries. Instructions for data upload or use can be found online (https://www.sharkipedia.org/procedure).

## Data Availability

The code for the creation and deployment of the database can be found online at www.github.com/sharkipedia/sharkipedia.
